# p62/SQSTM1 Accumulation in Squamous Cell Carcinoma of Head and Neck Predicts Sensitivity to Phosphatidylinositol 3-Kinase Pathway Inhibitors

**DOI:** 10.1371/journal.pone.0090171

**Published:** 2014-03-05

**Authors:** Wen-Liang Kuo, Marina N. Sharifi, Mark W. Lingen, Omar Ahmed, Jing Liu, Madhavi Nagilla, Kay F. Macleod, Ezra E. W. Cohen

**Affiliations:** 1 Department of Medicine, University of Chicago, Chicago, Illinois, United States of America; 2 Committee on Cancer Biology, University of Chicago, Chicago, Illinois, United States of America; 3 Ben May Department for Cancer Research, University of Chicago, Chicago, Illinois, United States of America; 4 Department of Pathology, University of Chicago, Chicago, Illinois, United States of America; 5 Comprehensive Cancer Center; University of Chicago, Chicago, Illinois, United States of America; Johns Hopkins Medical School, United States of America

## Abstract

The phosphoinositol-3 kinase (PI3K) pathway is highly dysregulated in squamous cell carcinoma of the head and neck (SCCHN). While inhibitors of the PI3K/AKT pathway are being developed in cancer, their efficacy does not appear to be related to the presence of mutations or amplification in pathway genes. The PI3K pathway is a major regulator of macro-autophagy, an evolutionarily conserved catabolic process that degrades cellular materials to promote cellular homeostasis and survival under stress. Employing a panel of SCCHN cell lines, we observed a significant correlation between the activity of PI3K/AKT inhibitors and their ability to induce autophagy. More specifically, resistance to these inhibitors was associated with accumulation of p62/SQSTM1, a pleotropic protein that is consumed during autophagy, while loss of autophagy was, for the first time, found to be due to silencing of an essential autophagy gene, ATG7. Moreover, modulating ATG7 and p62/SQSTM1 could regulate sensitivity to PI3K/AKT inhibitors, underscoring a mechanistic link between autophagy and drug sensitivity. Analysis of human tissues revealed progressive accumulation of p62/SQSTM1 in a significant proportion of cancer samples compared to normal tissue, suggesting that defective autophagy has relevance to SCCHN. These findings are further validated by analysis of TCGA data confirming homozygous deletion and mRNA down-regulation of *ATG7* in 10.0% of SCCHN samples. Taken together, these data indicate that p62/SQSTM1 levels modulate sensitivity to PI3K/AKT inhibitors; cancers vary in their capacity to undergo autophagy through epigenetic modification and, when deficient, accumulate p62/SQSTM1; and expression of autophagy-related proteins may serve as markers for resistance to PI3K/AKT inhibitors in SCCHN.

## Introduction

The phosphotidylinositol-3 kinase (PI3K) signaling pathway is a key regulator of cellular growth and stress responses that is constitutively activated in many cancers [1]. Specific mutations or copy number variations in PI3K pathway components, in addition to other pathway alterations have been discovered in almost every human malignancy analyzed [2], [3]. These findings have driven the development of PI3K pathway inhibitors that include specific inhibitors of PI3K subunit 3, specific AKT inhibitors as well as inhibitors of mTORC1 and mTORC2 [4], [5]. Paradoxically, despite the relative success of some of these pathway inhibitors in clinical trials, alterations in the pathway are neither sufficient nor necessary for response to these agents and reliable biomarkers that predict successful therapeutic efficacy for these agents have been lacking [6], [7].

Squamous Cell Carcinoma of the Head and Neck (SCCHN) is the 6^th^ most common malignancy worldwide [8] with a worldwide incidence of at least 500,000 and will be diagnosed in approximately 45,000 new patients in the United States this year [9]. Furthermore, population data from the United States demonstrate that some types of SCCHN, those associated with Human Papillomavirus infection, have been dramatically increasing in incidence in recent years [10], indicating that SCCHN is likely to become a yet more pressing health challenge in the future. It is now clear from DNA sequencing and gene copy number data that SCCHN tumors harbor amongst the highest rate of PI3K pathway genomic alteration of any malignancy [11]–[15]. Inhibitors of this pathway, therefore, have promise in SCCHN and are being actively developed.

Macro-autophagy has recently emerged as a major cellular process regulated by PI3K signaling that affects response to PI3K/AKT/mTOR inhibitors in both mouse models of cancer and in primary human cancers [5], [16]. Autophagy is an evolutionarily conserved catabolic process whereby cells degrade and recycle aggregated protein complexes, poorly functioning organelles and pathogens allowing cells to survive starvation and other stresses [17], [18]. The role of macro-autophagy (henceforth referred to as autophagy) in tumorigenesis and therapy responsiveness is complex, as it appears to both promote and inhibit tumor growth and progression, depending on stage of progression, driving oncogene and tissue type [19], [20].

This work shows that the sensitivity of squamous cell carcinoma cell lines to PI3K and AKT inhibitors is heavily influenced by the ability to undergo functional macro-autophagy and that sensitivity can be regulated by modulating autophagy related genes. We identify loss of ATG7 expression as a means to explain both abrogated macro-autophagy and increased resistance to PI3K pathway inhibitors. The effect of ATG7 silencing and autophagy inhibition on sensitivity to PI3K inhibitors results in accumulation of p62/SQSTM1, which is associated with increased anti-oxidant response and tumor cell survival and, in fact, increased p62/SQSTM1 expression is observed in primary SCCHN tumors. These results emphasize the importance of understanding the unique role played by macro-autophagy in specific tumor types and in response to key therapeutic interventions for each cancer.

## Materials and Methods

### Cell lines and reagents

CAL27, Detroit 562 (CCL138), and HEK293t cell lines were purchased from American Tissue Culture Collection (Manassas, VA), SQ-20B, SCC25, SCC35, SCC28, SCC58, and SCC61 cell lines [21] were provided by Dr. Ralph Weichselbaum, University of Chicago. HN5 cells [22] were provided by the Ludwig Institute for Cancer Research (London, UK). Breast cancer cell lines, HCC38, T47D, MDA-MB468, HCC1937, SKBR3, MCF-7, MDA-MB231 and HS578T, were provided by Dr. Kay Macleod, University of Chicago and were originally purchased from American Tissue Culture Collection (Manassas, VA). All cell lines were cultured in culture medium containing 10% fetal bovina serum and Pen/Strep (concentration).

Phospho-Akt (Ser473), phospho-Akt (Ser308), phospho-S6 Kinase (Ser254/256), phospho-GSK-3beta (Ser9), LC3B (#3868), ATG7 (#2631), Beclin-1 (#3495), ATG5 (#8540), ATG12 (#4180), and Rapamycin were purchased from Cell Signaling Technology Inc (Danvers, MA). p62/SQSTM1 (PM045) antibody was purchased from MBL Co. Antibodies against alpha-tubulin (sc-8035) were purchased from Santa Cruz Biotechnology (Santa Cruz, CA), Cyclin D1 (NB 600-584) from Novus Biologicals, Inc. (Littleton, CO), and HA rat monoclonal antibody (3F10) from Roche Applied Science (Indianapolis, IN). IRDye 800 anti-mouse IgM Mu chain specific antibody was purchased from Rockland Immunochemical for Research (Gilbertsville, PA). Other IRDye secondary antibodies were purchase from Li-Cor Biosciences (Lincoln, NE). Doxycycline Hydrochloride (#BP2653-1) was purchased from Fisher Scientific. SAR245408 and SAR245409 were provided by Sanofi-Aventis Bridgewater, NJ, and MK-2206 was provided by Merck & Co., Inc., Rahway, NJ.

### ShRNA-p62/SQSTM1 lentiviral vectors and siRNA transfection

A set of p62/SQSTM11 shRNA vectors (SHCLNG-NM_003900), and a non-target shRNA control vector were purchased from Sigma-Aldrich (St. Louis, MO) for the p62/SQSTM1 knockdown experiments. Lentivirus production and viral infection were done as previously described [23]. Small interfering RNA (siRNA) duplexes were purchased from Ambion (Austin, TX) targeting ATG7 (ID# s20650 and s20651). Negative Control #1 siRNA was used as control. siRNAs (30 µM per 100 µl transfection solution) were electroporated using Lonza's Nucleofector and the Cell Line Neucleofection kit V (Walkersville, MD) As described in [23]

### Expression of p62/SQSTM1 in SCC61 and MCF-7 cells

The vector expressing HA-tagged p62/SQSTM1, pLenti-GIII-CMV-hSQSTM1-HA, and Lenti-combo packing mix purchased from Applied Biological Materials Inc. (Richmond, BC, Canada) were used to transect into 293T cells to produce p62/SQSTM1-HA lentiviruses for transduction of SCC61 cells. SCC61 cells stably expressing p62/SQSTM1-HA were selected in growth medium containing 4 µg/ml of puromycin. For overexpression of p62/SQSTM1 in MCF-7 cells, pLenti-GIII-hSQSTM1-HA vector DNA was transfected with TransIT-LT1 (Mirus Bio LLC, Madison, WI) into MCF-7 cells and the transiently transfected cells were used for assays.

### Establishing inducible knockdown of *ATG7* in SCC61 cells

The pTRIPZ-hAtg7shRNA vector, a Tet-On expression vector, the pGIPZ-non-Silencing control vector, and Trans-Lentiviral packaging mix were purchased from Fisher Scientific Company (Hanover Park, IL). Production of lenti-viruses, lentiviral transduction, and selection of puromycin-resistant SCC61 cells were done as described above. The Tet system approved fetal bovine serum (Clontech Laboratories, Inc, Mountain View, CA) was used in culturing the stable SCC61 cell lines and Doxycycline was added to cell culture (1 µg/ml of final concentration) to induce the expression of hAtg7 shRNA in cells.

### Establishing SCC35-mycAtg7 cell line

A lentiviral vector expressing human *ATG7* gene was constructed by ligating the EcoRI-NotI fragment containing myc-hAtg7 from pCMV-myc-Atg7 vector [24] into pCDH-CMV-MCS-EF1-puro vector. Lentiviruses expressing mycAtg7 were produced as described above. Transduction of SCC35 cells and selection of selection of SCC35-mycAtg7 cell line are previously described [23].

### Bisulfite conversion and methylation-specific PCR

Genomic DNA was isolated using Wizard SV Genomic DNA Purification System (Promega). Bisulfite conversion of genomic DNA was done with EpiTect Fast Bisulfite kit (Qiagen) following the manufacture's recommended protocol. Methylation-specific PCR was done with HotStar d-Tect polymerase (EpiTect MSP Kit from Qiagen) and the methylation-specific, unmethylated-specific, and wild-type primer sets as described in Table S6 in File S1. PCR condition was 95°C for 10 minutes, 35 cycles of 94°C for 15 seconds, 55°C for 30 seconds, 72°C for 30 seconds, and finished with an extension at 72°C for 10 min. The Methyl Primer Express Software v1.0 (Life Technologies, Inc) was used to predict CpG island and to design PCR primers.

### Cell cycle analysis and cell viability assay

Cell cycle analysis was done with Propidium Iodide (PI) staining as described in [23]. For cell viability assay, 1000–2000 cells were seeded in 96-well dishes, and the cells were treated with drugs and assayed with CellTiter-Blue reagent (Promega, Madison, WI) as described in [23]. GraphPad or Grafit Software was used to calculate IC50s.

### Protein immunoblot analysis

Cell lysis, protein concentration, separation, and transfer were determined as previously described [23]. Membranes were blocked using blocking buffer (Li-Cor Biosciences, Lincoln, NE). Secondary antibodies were infrared-labeled. Odyssey Infrared Imaging system (Li-Cor Biosciences, Lincoln, NB) was used for protein detection and quantification.

### Quantitative real-time PCR

RNA was isolated as previously described [23]. *ATG7* RNA was measured by *ATG7* primers TaqMan RNA assay (Applied Biosystems) and determined by qRT-PCR performed on a StepOnePlus machine (Applied Biosystems).

### Tissue Microarrays (TMA)

Custom human tissue microarrays (TMA) were fabricated in the Human Tissue Resource Center of the University of Chicago. Serial sections were prepared from TMA blocks containing normal, dysplastic, and malignant head and neck tissues cores. The sections were stained with antibodies against phospho-specific AKT, p62/SQSTM1, and ATG7 proteins separately. The stained slides were scanned at 20× using the Aperio ScanScope XT instrument and were analyzed in ImageScope viewing software using Genie and a Color Deconvolution macro (a macro is a modified algorithm). Genie is a pattern recognition algorithm that was trained to recognize tumor cells present in the tissue. The Genie macro was applied to the Color Deconvolution macro to analyze the intensity of tumor cells, ignoring all other cellular types present in tissue.

## Results

### SCCHN tumor cells exhibit varying degrees of sensitivity to PI3K/AKT inhibitors that does not correlate with PIK3CA mutational status

**Figure 1:**
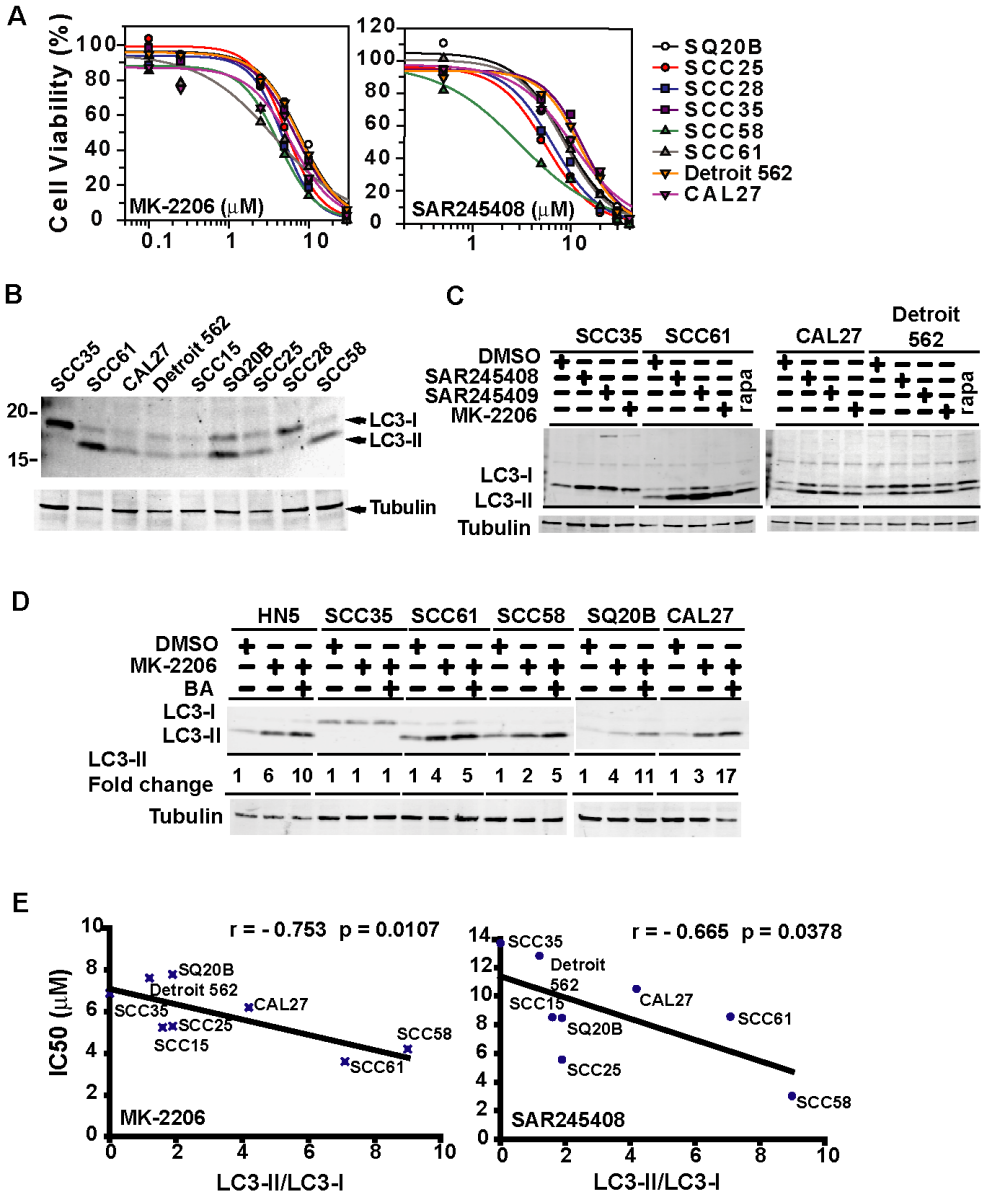
Autophagy is induced in SCCHN cell lines by MK-2206 and SAR245408 and autophagy competence positively correlates with drug sensitivity. **A**) Dose-response curve of SCCHN cell lines to MK-2206 and SAR245408. Cells were treated with the inhibitors for 72 hours and cell viability were assayed using Cell-Titer Blue reagent as described in Methods. **B**) SCCHN cell lines show variable autophagic activity defined by the ratio of LC3-II/LC3-I. The intensity of LC3-I and LC3-II bands were quantified with a Li-Cor imaging scanner. **C**) Western blots show PI3K/mTOR inhibitors induce autophagy in several SCCHN cell lines but not in SCC35 cells. **D**) Effect of MK-2206 and bafilomycin A1 on LC3-II protein in SCCHN cell lines. Cells were treated for 24 hours and bafilomycin A1 (final concentration 100 nM) was added 4 hours before harvesting. Western blotting was probed with LC3 antibody. LC3-II was quantified and normalized to tubulin. **E**) Drug sensitivity to MK-2206 and SAR245408 correlated with intrinsic autophagy competence.

Activating mutations in *PIK3CA* have been hypothesized to predict responsiveness to PI3K inhibitors and there are at least two SCCHN cell lines, SCC61 (E542K) and Detroit 562 (H1047R), which harbor such mutations [25]. However, when we screened a panel of SCCHN cell lines for sensitivity to increasing concentrations of SAR245408, a selective class I PI3K inhibitor, and MK-2206, an allosteric AKT inhibitor, we observed that *PIK3CA* mutational status did not robustly predict drug sensitivity (Figure 1A, Table S1 in File S1). In fact, the Detroit 562 cell line was relatively resistant to PI3K pathway inhibitors despite harboring a *PIK3CA* mutation suggesting that additional factors contribute to the sensitivity of tumor cells to this class of agents. Nonetheless, MK-2206 and SAR245408 specifically and efficiently inhibited downstream effectors of PI3K, AKT and S6 in File S1, in SCCHN cells (Figure S1-A in File S1). Thus resistance of SCCHN cells to the drugs cannot be explained by lack of putative target inhibition. Furthermore, apoptotic cells represented no more than 10% of the total cell population upon exposure to MK-2206 and SAR245408 (Table S2 in File S1) indicating that apoptosis induction did not contribute to the differences in drug sensitivity. All cell lines also showed reduced cell cycle progression in response to drug (Figure S1-B in File S1) as well as reduced cyclin D1 expression in an equivalent and transient manner (Figure S2 in File S1). These results indicate that neither apoptosis induction nor cell cycle differences are determinants of sensitivity to PI3K pathway inhibitors in SCCHN cell lines and suggest that other biological processes are more relevant.

### Sensitivity to PI3K/AKT Inhibitors is associated with induction of macro-autophagy

Induction of macro-autophagy is another phenotypic response to PI3K pathway inhibitors [26], [27]. To assess whether differences in the ability to undergo functional autophagy explained the observed differential sensitivity of SCCHN cell lines to SAR245408 and MK2206, we first examined LC3B, a commonly used marker of autophagosome formation and autophagic flux [28]. Interestingly, we observed marked differences in baseline autophagic activity between different SCCHN cell lines (Figure 1B). For example, the SCC35 cell line showed very low levels of processed LC3B-II (Figure 1B, lane 1) while SCC61 cells showed a much higher level of processed LC3B-II (Figure 1B, lane 2). These effects were exaggerated when cells were exposed to SAR245408, MK2206, or SAR245409, a dual inhibitor of PI3K and mTOR (Figure 1C). To examine whether the cell lines are autophagy competent, we measured autophagy flux by treating cells with bafilomycin A (BA) before harvesting, and we showed that increased levels of processed LC3B-II in some SCCHN cell lines (HN5, SCC61, SQ20B, CAL27, and SCC58) following drug treatment except SCC35. Furthermore, the results reflected increased autophagic flux since pre-treatment of cells with bafilomycin A (BA) increased the overall levels of processed LC3B-II compared to drug treatment alone (Figure 1D). These results demonstrate that MK-2206 induced autophagic flux to some degree in all cell lines except SCC35. Although the fold increase in autophagy flux were higher in cell lines such as HN5, SQ20B, and CAL27 compared to SCC61 and SCC58, the higher fold increase can be mostly attributed to low baseline level of LC3B-II. We analyzed the correlation between baseline levels of processed LC3B-II (Figure 1B) and the IC_50_ of each cell line for PI3K pathway inhibitors and found a negative linear association (Table S3 in File S1, Figure 1E).

**Figure 2:**
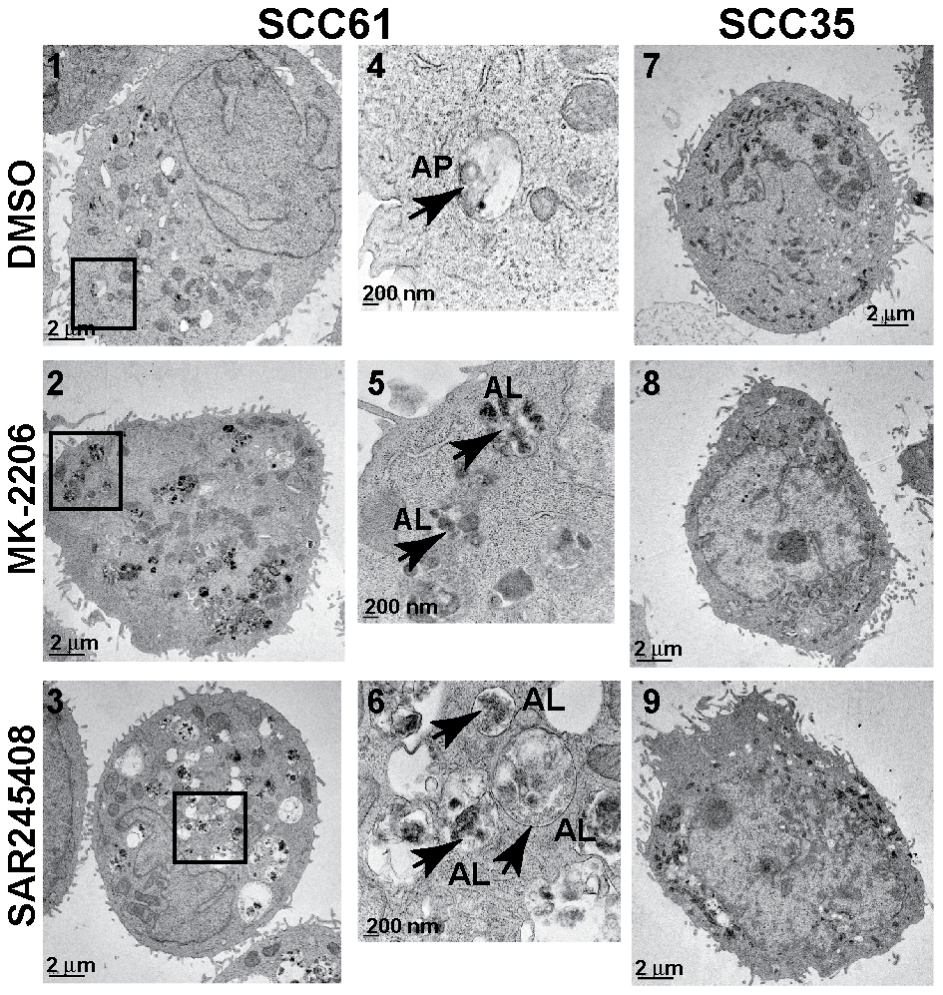
Induction of autophagy by MK-2206 and SAR245408 in SCC61 cells, not in SCC35 cells, is shown by transmission electron microscopy. Cells were treated with the inhibitors for 24(AP) and autophagolysosomes (AL) are indicated by arrows.

Induction of autophagy by MK-2206 and SAR245408 was further verified by transmission electron microscopy (TEM) where we detected a significant increase in large cargo-laden autophagosomes and autophagolysosomes following treatment with either inhibitor of the sensitive SCC61 cell line (Figure 2, panels 1–6) compared to treatment with vehicle control (DMSO). In contrast, we failed to detect any autophagosomes before or after treatment of the inhibitor-resistant SCC35 cell line (Figure 2, panels 7–8).

**Figure 3:**
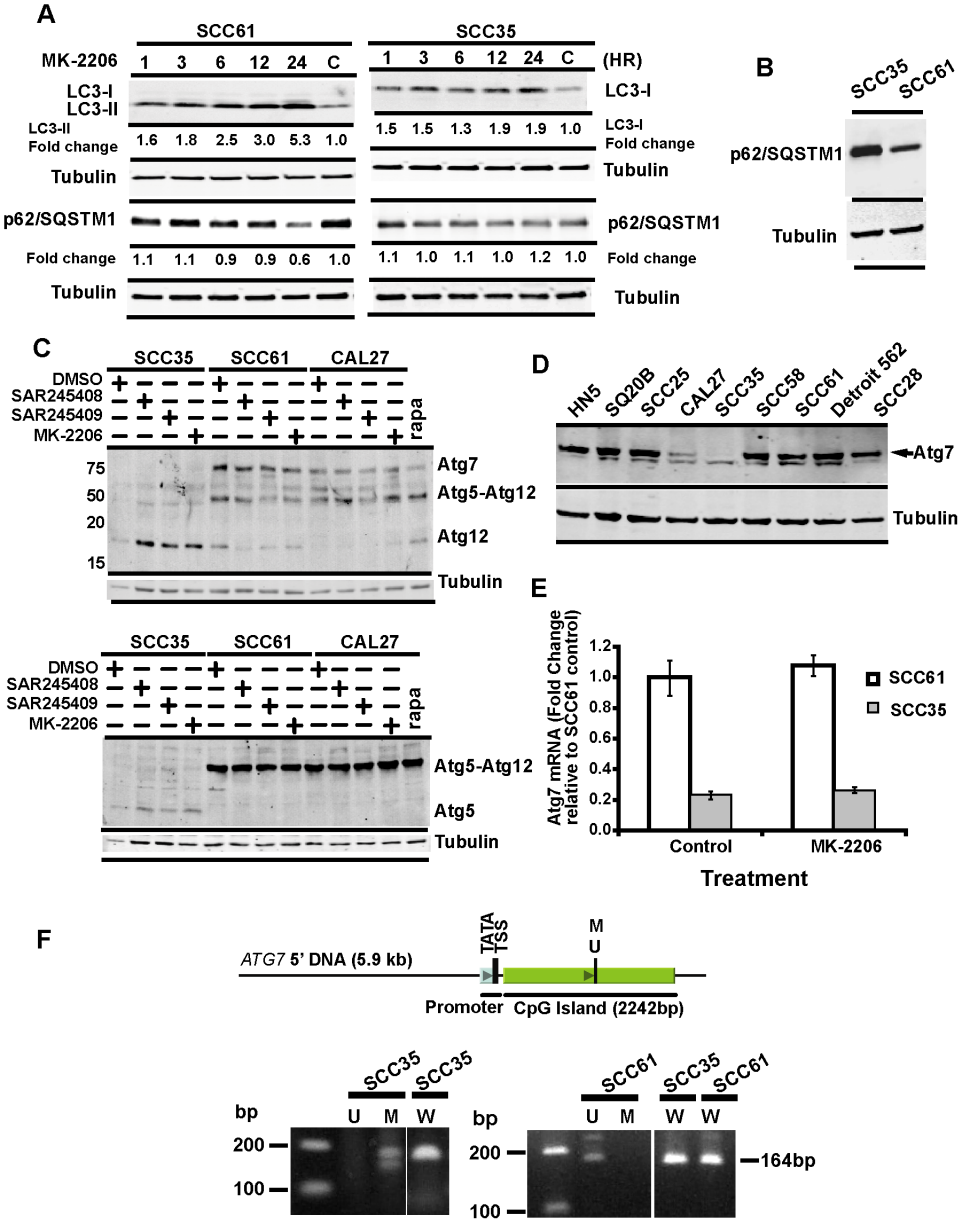
SCC35 cell line is deficient in ATG7 expression and is macro-autophagy deficient. Time course of autophagy induction by MK-2206 in SCC61 (**A**) and SCC35 (**B**) cells. Western blots show LC3-II and p62/SQSTM1 levels from cells treated with MK-2206 (5 µM) at the time points indicated. Fold change was calculated by the ratio of the MK-2206 treated to untreated controls (C). **C**) Formation of ATG5-ATG12 conjugates in SCCHN cell lines. Western blotting was probed sequentially with ATG12 and ATG7 antibodies (top). The same lysates were analyzed with an ATG5 antibody (bottom). **D**) ATG7 protein expression in a panel of SCCHN cell lines by western blotting. A non-specific band was detected below the ATG7 protein. **E**) Detection of *ATG7* mRNA by Real-time PCR in both untreated (control) and MK-2206 treated SCC35 and SCC61 cells. **F**) Methylation-specific PCR amplification of the 5′ untranslated region of *ATG7* gene in SCC35 and SCC61 cells. Untreated and bisulfite treated SCC35 and SCC61 genomic DNAs were used as templates for PCR amplification. The PCR products were analyzed on a 1.2% agrose gel. The unmodified genomic DNA was used with the wild type primers and bisulfite treated genomic DNAs were used for both unmethylated and methylated specific primers. The primer region contains 2 CpG sites as listed in Methods.

Expression of p62/SQSTM1 has also been utilized as an indirect measure of autophagy in some cell types since it is a multifunctional adaptor protein that serves to target certain cytoplasmic cargo to the autophagosome and is consumed during autophagic flux [29], [30]. Thus, p62/SQSTM1 expression decreases during autophagy induction. Consistently, we observed that p62/SQSTM1 levels decreased as processed LC3B-II levels increased in drug-sensitive SCC61 cells in response to treatment with MK2206 (Figure 3A, left). By contrast, no change in p62/SQSTM1 expression was detected in the drug-resistant SCC35 cells (Figure 3A, right) and indeed SCC35 cells expressed higher basal levels of p62/SQSTM1 compared to drug-sensitive SCC61 cells consistent with a defect in autophagy (Figure 3B). These results suggest that autophagy deficiency in SCC35 cells may lead to p62/SQSTM1 accumulation and contribute to resistance to PI3K pathway inhibitors.

### Defective autophagy in drug-resistant SCCHN cells is associated with loss of ATG7 expression

To gain insight into the mechanism of autophagy deficiency in SCC35 cells, we measured levels of ATG7 and ATG5-ATG12 conjugation in different SCCHN cell lines since ATG7-mediated conjugation of ATG12 to ATG5 is an essential step in autophagosome formation. We observed robust ATG7 expression and formation of the ATG5-ATG12 conjugate in drug sensitive SCC61 cells. However, ATG7 protein was undetectable in the resistant SCC35 cells consistent with loss of ATG7 expression (Figure 3C), even though unconjugated ATG5 and ATG12 were readily detected. Indeed, the absence of ATG7 in SCC35 cells is consistent with their inability to undergo effective LC3 conversion (Figure 3A right) and this being the underlying mechanism of defective autophagy. By contrast, the more sensitive SCC61 cell line underwent efficient LC3 processing (Figure 3A, left).

ATG7 protein expression was undetectable in SCC35 cell line (Figure 3D, lane 5) and RT-PCR demonstrated that *ATG7* mRNA in SCC35 cells was also dramatically reduced compared to SCC61 cells (Figure 3E) suggesting reduced transcription of *ATG7* in drug resistant SCC35 cells. We hypothesized that *ATG7* was being epigenetically silenced in SCC35 cells and, using Methyl Express Primer software, we identified a CpG island near the *ATG7* promoter (Figure 3F, top). Indeed, methylation-specific primers revealed a CpG-region of the *ATG7* promoter that is hypermethylated in SCC35 but unmethylated in SCC61 cells (Figure 3F, bottom) supporting our hypothesis that transcriptional inactivation of *ATG7* in SCC35 cells is likely due to epigenetic silencing of the *ATG7* promoter.

### Regulation of ATG7 modulates PI3K/AKT inhibitor sensitivity

**Figure 4:**
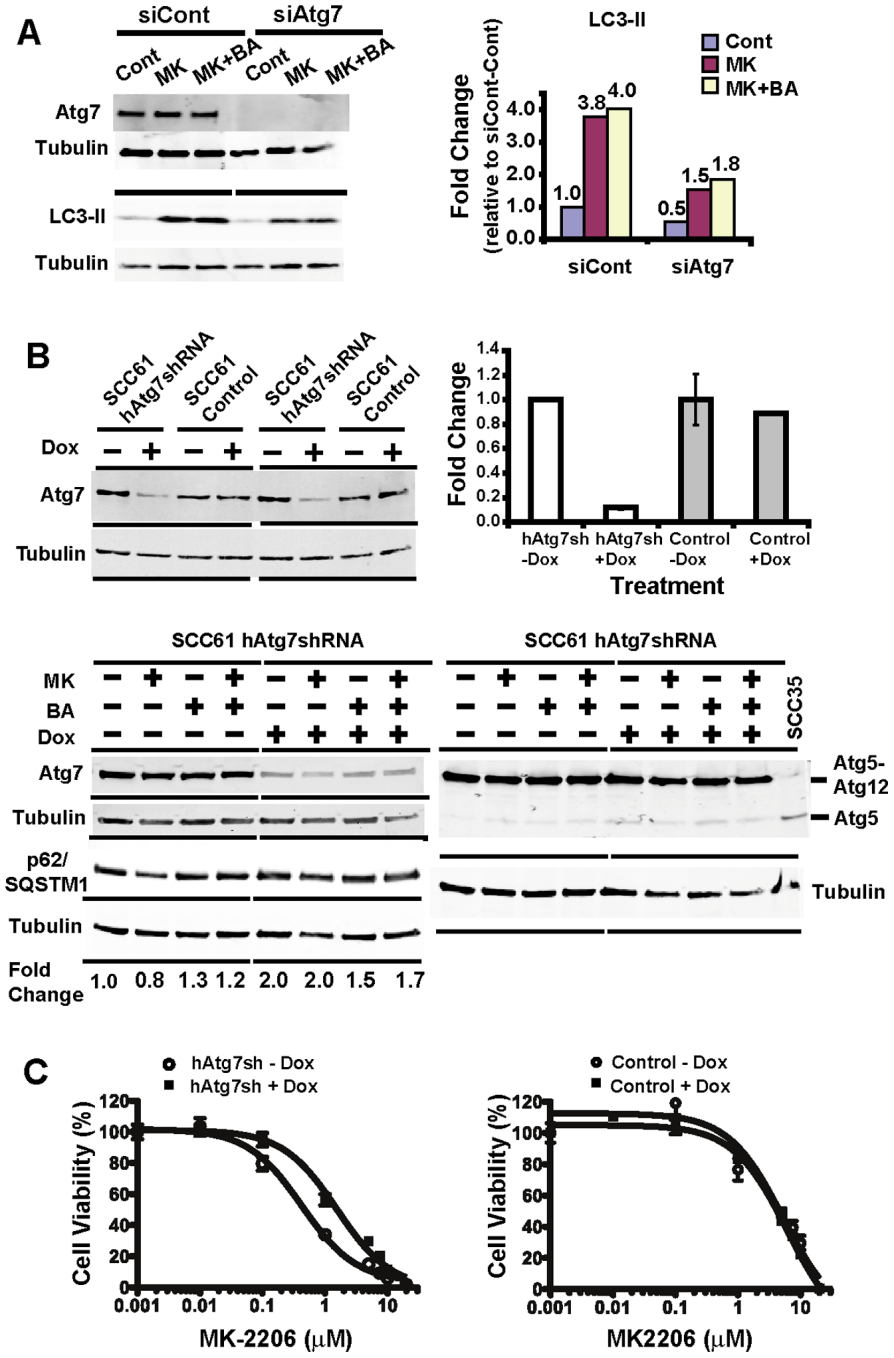
Regulation of ATG7 modulates drug sensitivity in SCC61. **A**) Depletion of ATG7 protein expression in SCC61 cells reduced LC3-II. SCC61 cells were transfected with control siRNA or ATG7 siRNA and analyzed by western blotting. LC3-II protein was quantified and the results shown graphically. **B**) Generation of conditional knockdown of ATG7 in SCC61-hAtg7shRNA cells. Doxycycline (1 µg/ml) was added for a week to knockdown ATG7 expression. Western blotting for ATG7 expression and graphic representation of quantified ATG7 are shown (top panel). The SCC61-hAtg7shRNA cells were treated with MK-2206 and Bafilomycin as described in Methods. Western blots show ATG7, ATG5, ATG5-ATG12 conjugates, and p62/SQSTM1 protein. p62/SQSTM1 quantification was normalized to tubulin and fold change was compared to untreated control (first lane). **C**) ATG7 depleted SCC61 cells are significantly more resistant to MK-2206. Cell viability assays were preformed in the presence of MK-2206 (5 µM) or the vehicle control (DMSO).

ATG7 plays a critical role in canonical autophagosome formation and its depletion is predicted to effectively abrogate autophagy, unless alternative sources of phagophore membranes are utilized [31], [32]. In order to determine whether effective autophagy was critical to PI3K and AKT inhibitor sensitivity, we depleted ATG7 in the more sensitive SCC61 cells using siRNA and an inducible Atg7shRNA lentiviral vector. When *ATG7* is significantly depleted in SCC61 cells, the LC3-II level is dramatically reduced (Figure 4A). However, stable knockdown of *ATG7* with constitutively expressed Atg7shRNA in SCC61 cells was incompatible with cell survival (data not shown). We therefore utilized an inducible vector, pTRIPZ-hAtg7shRNA, and established a stable SCC61 cell line where induced expression of hAtg7shRNA with doxycycline significantly reduced ATG7 protein (>80%) compared to untreated controls (Figure 4B, top). Induced ATG7 depletion resulted in increased free ATG5 in cells treated with doxycycline (Fig. 4B, bottom-right). In addition, bafilomycin A1 treatment increased p62/SQSTM1 protein expression (Figure 4B, bottom-left) consistent with a block in autophagolysosome formation and, more importantly, p62/SQSTM1 expression concomitantly increased in ATG7 depleted cells. Taken together, these results support that knockdown of ATG7 in SCC61 cells significantly disrupted autophagy. Significantly, doxycycline treated SCC61 cells were significantly more resistant to MK-2206 compared to no doxycycline control with IC_50_s of 1.56 µM and 0.43 µM, respectively (Figure 4C). These results demonstrate that inhibition of autophagy in SCC61 cells promotes drug resistance and indicates that autophagy is required for sensitivity to PI3K/AKT inhibitors.

### Regulation of p62/SQSTM1 modulates PI3K/AKT inhibitor sensitivity

**Figure 5:**
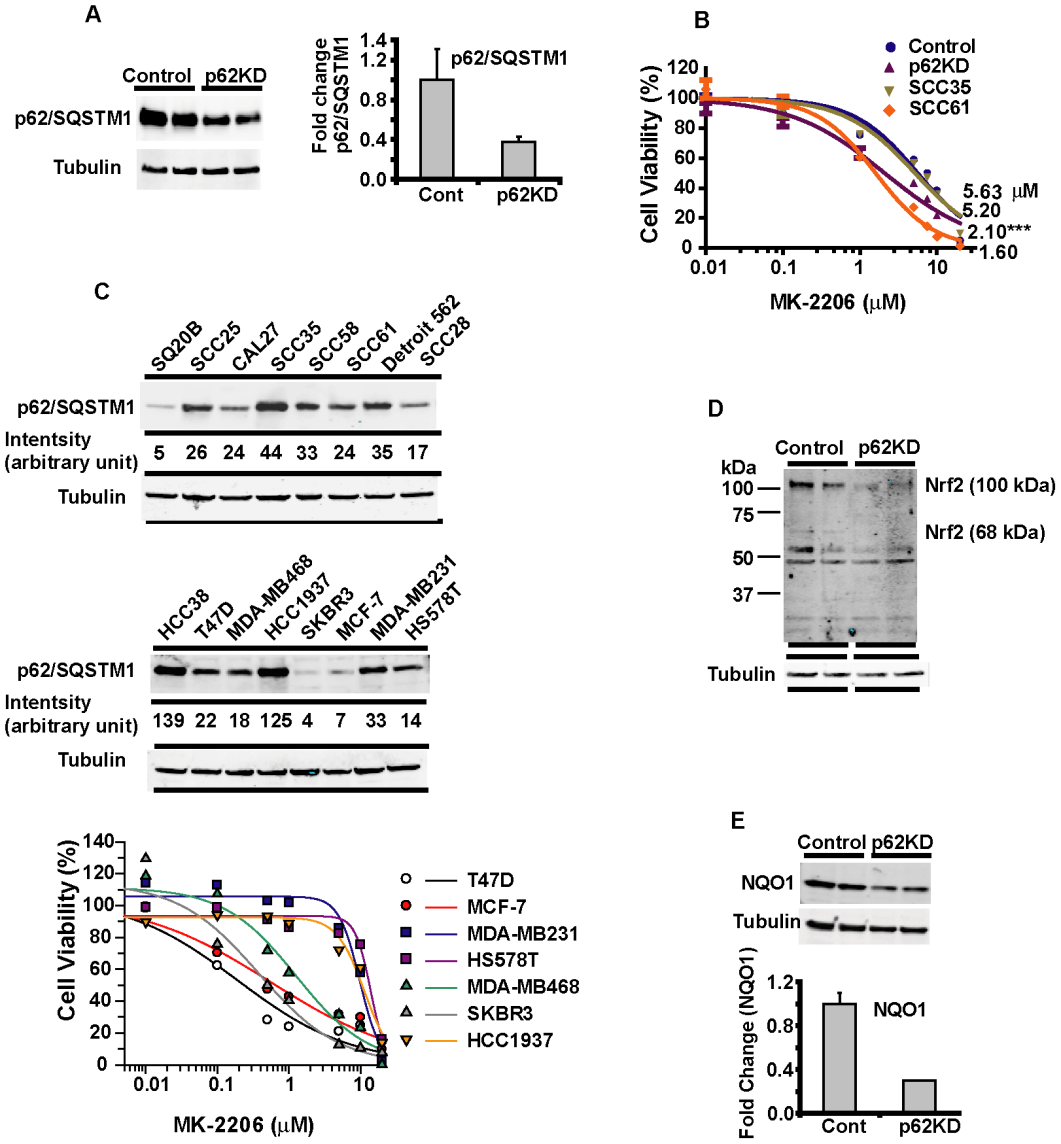
Effects of down regulation of p62/SQSTM1 in SCC35 cells and association of p62/SQSTM1protein level with drug resistance in both SCCHN and breast cancer cells. **A**) Western blotting shows knockdown of p62/SQSTM1 in SCC35-p62KD cell line compared to SCC35-control (left). The p62/SQSTM1 bands were quantified and shown graphically on the right. **B**) Dose-response curves of SCC35-p62KD, SCC35-control, parental cells (SCC35), and SCC61 cells to MK-2206. **C**) Detection of p62/SQSTM1 protein expression in panels of SCCHN (Top) and breast cancer cell lines (Middle) by western blotting as described in Methods. Dose-responsive curves of the panel of breast cancer cell lines treated with MK-2206 (Bottom). **D and E**) Reduced expression of Nrf2 (**D**) and NQO1 (**E**) in SCC35-p62KD and SCC35-control cells. Western blotting shows duplicate samples from each derived cell line. Graph represents quantified fold change normalized to control.

As demonstrated above and previously described [30], [33], p62/SQSTM1 is consumed when cells undergo autophagy. Thus we postulated that autophagy prevented resistance to PI3K pathway inhibitors by promoting p62/SQSTM1 turnover. We investigated the role of p62/SQSTM1 in drug resistance in SCCHN by altering expression of p62/SQSTM1. Stably depleting p62/SQSTM1 in SCC35 derived cells, SCC35p62KD, resulted in a 60–70% reduction in p62/SQSTM1 protein expression (Figure 5A) which led to a significant increase in sensitivity of these cells to MK-2206 compared to SCC35-Control cells expressing the control scrambled shRNA (Figure 5B). Importantly, SCC35p62KD cells now showed similar sensitivity to MK2206 as drug sensitive SCC61 cells (Figure 5B). These results are consistent with the hypothesis that relatively high expression of p62/SQSTM1, arising in SCC35 cells secondary to a defect in autophagy, drives resistance to PI3K pathway inhibitors.

**Figure 6:**
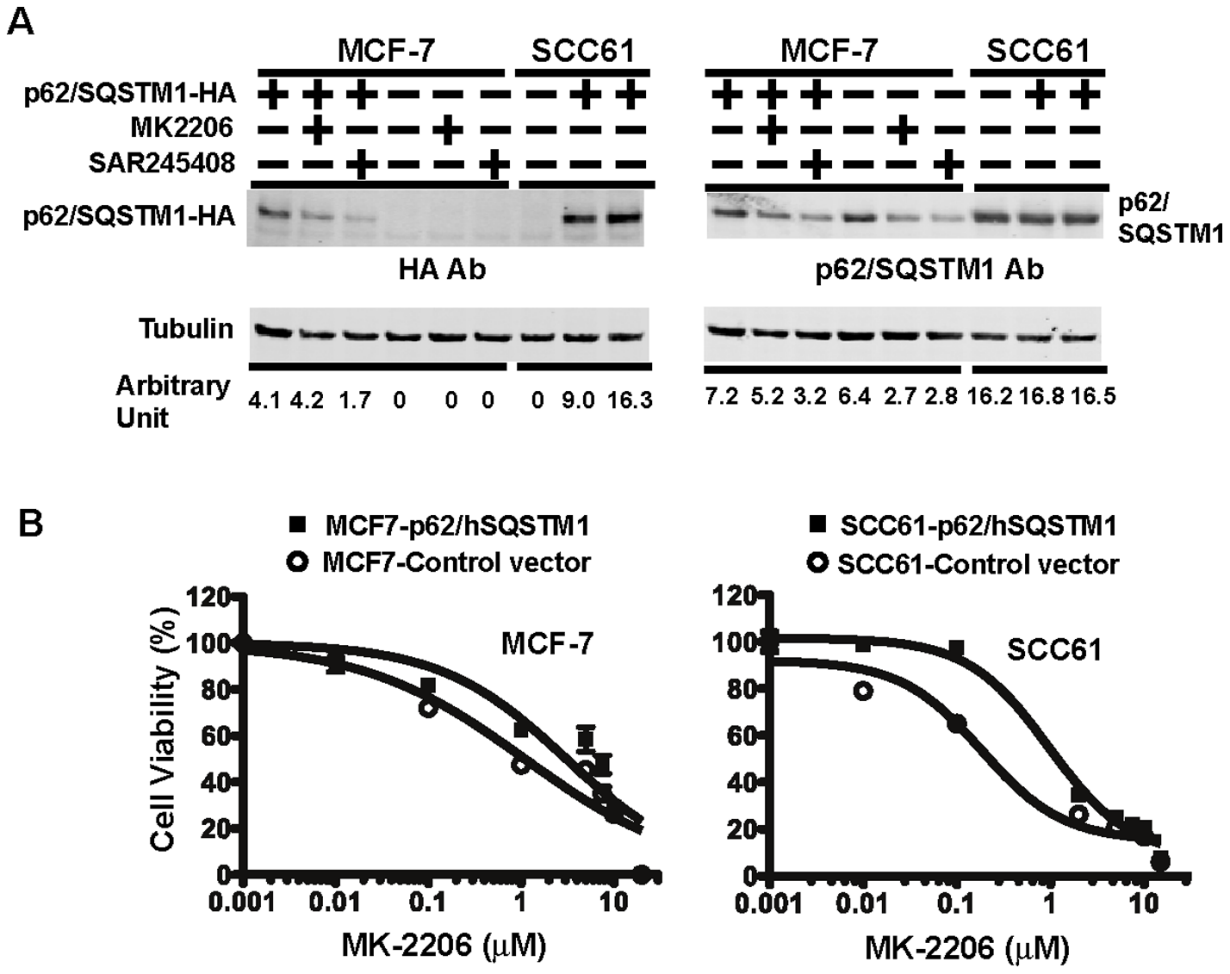
Overexpression of p62/SQSTM1 increases drug resistance in both SCCHN and breast cancer cells. **A**) MCF-7 cells were transiently transfected with p62/SQSTM1 expressing vector and SCC61 cells were stably transduced with lentivirus carrying the same p62/SQSTM1 vector. Western blotting shows the ectopically expressed p62/SQSTM1-HA protein detected with HA antibody (3F10). The same blot was reprobed with p62/SQSTM1 antibody to show the total p62/SQSTM1 protein expression in both cell types. **B**) Cell viability assays demonstrate that p62/SQSTM1 expression increases drug resistance in both MCF-7 and SCC61 cell lines.

We then asked whether p62/SQSTM1 protein expression is a predictive marker for PI3K pathway inhibitors in SCCHN cell lines. We determined the expression of p62/SQSTM1 protein in SCCHN cell lines (Figure 5C, top) and found a positive correlation with sensitivity to the PI3K inhibitor SAR245408 and a similar trend to MK-2206 (Table S4 in File S1). To test these findings in other tumor cell types, we expanded our analysis to a panel of breast cancer cell lines where we detected a range of p62/SQSTM1 protein expression (Figure 5C, middle) and drug sensitivity to MK2206 (Figure 5C, bottom). Interestingly, the positive correlation found in SCCHN cell lines between p62/SQSTM1 expression and PI3K/AKT inhibitors sensitivity was also observed in breast cancer cells (Table S5 in File S1). These results indicate that resistance to PI3K and AKT inhibitors is associated with higher levels of p62/SQSTM1 in both SCCHN and breast cancer cells. We then investigated mechanisms of resistance of PI3K pathway inhibitor mediated by p62/SQSTM1 in SCCHN cells. p62/SQSTM1 is a multi-functional adaptor protein that interacts with molecules involved in inflammation and oxidative stress [29], [34] in addition to autophagy. For example, p62/SQSTM1 stabilizes Nrf2 and promotes its transcriptional activation of antioxidant and detoxifying genes, such as NQO1 [35], [36]. Given that we observe accumulation of p62/SQSTM1 in autophagy-deficient and drug resistant SCCHN cell lines, we investigated whether expression of Nrf2-regulated genes was influenced by p62/SQSTM1 in these cell lines. Indeed, abrogated expression of p62/SQSTM1 reduced expression of both Nrf2 (Figure 5D) and its target gene, NQO1 (Figure 5E) in SCC35 cells compared to baseline. Conversely, ectopic expression of HA-tagged p62/SQSTM1 in relatively drug sensitive cell lines such as SCC61 and MCF-7 (Figure 6A) rendered these cells more resistant to MK-2206 compared to control (Figure 6B) Taken together, these results indicate that p62/SQSTM1 is a predictive marker of resistance to PI3K pathway inhibitors in SCCHN and breast cancer cell lines, and that autophagy deficiency can lead to increased expression of p62/SQSTM1, suggesting that elevation of p62/SQSTM1 in cells can promote resistance through increased Nrf2 activity and expression of Nrf2-regulated anti-oxidant genes.

### Expression of Autophagy Related Proteins in SCCHN Tissue

**Figure 7:**
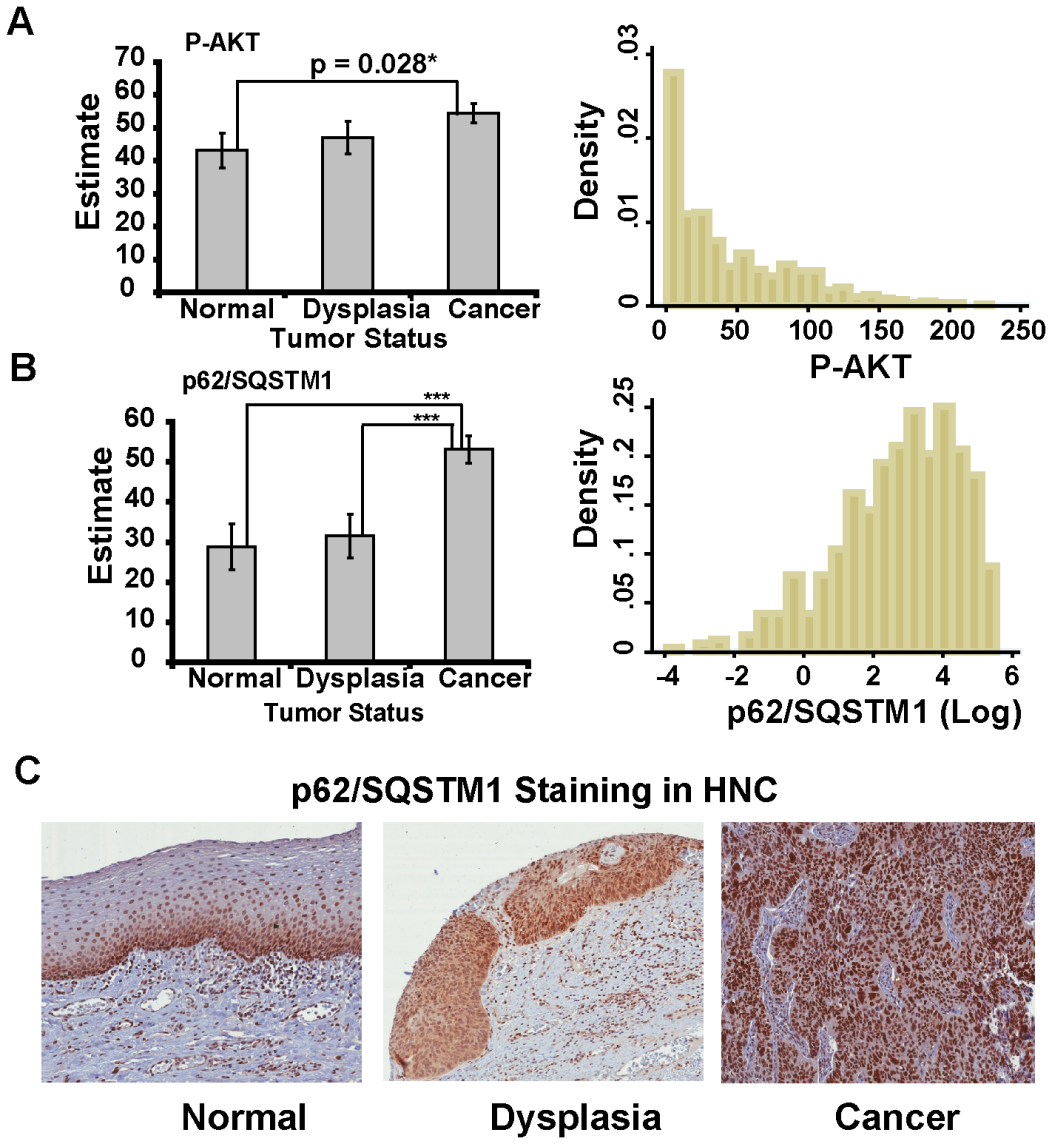
Expression of phospho-AKT and p62/SQSTM1 in tissue microarrays of normal, dysplastic, and malignant oral mucosa. **A**) Expression of phospho-AKT. Tissue microarrays were stained with phospho-AKT specific antibodies and scored as described in Methods. A total of 362 individual samples were analyzed which included 76 normal, 87 dysplasia, and 199 cancer samples. Histograms show score distribution from cancer samples. **B**) Expression of p62/SQSTM1. The same tissue microarrays were stained with p62/SQSTM1 antibody and scored as described in Methods. Histograms show score distribution from cancer samples. **C**) Images (20× magnification) of representative immunohistochemical staining in normal, dysplasia, and cancer tissues obtained from the same patient.

The data above support a mechanistic role for autophagy and p62/SQSTM1 in determining the response of a subset of SCCHN cells to PI3K pathway inhibitors. In order to determine whether this has relevance to primary human SCCHN, we created tissue microarrays consisting of normal (n = 76), dysplastic (n = 87), and malignant (n = 199) oral mucosal tissue and performed immunohistochemical staining for autophagy related proteins. As expected, AKT phosphorylation increased steadily from normal to dysplastic to oral cancerous tissues reflecting progressive activation of the PI3K pathway during malignant progression (Figure 7A). In conjunction with a rise in phospho-AKT, which would be expected to inhibit autophagy, an increase in p62/SQSTM1 was also observed in primary SCCHN cancer compared to normal and dysplastic tissues (Figure 7B and 7C). p62/SQSTM1 expression in cancers was present in both nucleus and cytoplasm consistent with the finding that p62/SQSTM1 protein contains two nuclear localization signals and a nuclear export signal and that the protein can shuttle between nucleus and cytoplasm [37]. Our data, therefore, support a model in which accumulation of p62/SQSTM1 in SCCHN cancers promotes resistance to PI3K pathway inhibitors.

## Discussion

This work demonstrates, for the first time, a relationship between autophagy deficiency and resistance to a specific drug class, namely PI3K pathway inhibitors. Interestingly, this resistance was accompanied by accumulation of p62/SQSTM1 and partially reversed by its depletion. Moreover, tumor cell lines that are autophagy competent were more sensitive to PI3K inhibition, which is also reversed by depletion of obligatory macro-autophagy proteins. The findings that SCCHN display increased p62/SQSTM1 expression compared to normal and dysplastic tissue suggests that these findings are clinically meaningful and that there are many cancers that will be inherently resistant to PI3K inhibition due to defects in autophagy. In fact, alterations of *ATG7* gene were detected in TCGA Head and Neck Squamous Cell Carcinoma samples as we analyzed the data set using the cBio Cancer Genomic Portal (cBioPortal) [38]. These alterations include three cases of putative homozygous deletion, 27 cases of mRNA down-regulation (one case overlapped with homologous deletion), and two cases of mRNA up-regulation (Figure S3-A in File S1). Interestingly, overall survival from cases with alterations in *ATG7* was lower than cases without alterations (Figure S3-B in File S1) with median survival of 15 versus 28 months, respectively (log rank p-value 0.048964). Unfortunately, p62/SQSTM1 protein expression data are not available precluding an analysis of the relationship to *ATG7* alterations. Taken together, these results support the hypothesis that autophagy competence and p62/SQSTM1 could be used to stratify certain cancers for their response to specific therapies. This is in contrast to other published work where autophagy is more generally shown to promote drug resistance [39] and underscores the multi-faceted role of autophagy in cancer.

The novel observation that ATG7 expression can be lost was unexpected. ATG7 is required during autophagosome formation at two critical points: formation of ATG5-ATG12 conjugates and incorporation of LC3 into the membrane. Consistently, loss of ATG7 expression in SCCHN cells was associated with defects in autophagy. Given that targeted deletion of Atg7 in mice results in perinatal lethality and tissue specific deletion causes marked defects in both liver and brain [40], it was initially surprising that SCC35 cells express nearly undetectable levels of ATG7. Given their altered metabolism and increased cellular dysfunction, it might have been predicted that tumor cells would be critically dependent on ATG7 and autophagy for homeostasis and survival. ATG7 has been shown to have functions that are independent of macro-autophagy, including potentiating non-apoptotic cell death by caspase-8 [41] and regulation of p53 activity [42] and it is possible that loss of ATG7 in tumors is selected for to prevent autophagy-independent functions. Interestingly, when we attempted to rescue autophagic competence in SCC35 cells by ectopically expressing *ATG7* (Figure S4 in File S1), we could not induce autophagy or reverse drug resistance. The resistance phenotype, in fact, was only reversed by abrogating p62/SQSTM1 expression, which, in turn can be regulated by several processes beyond autophagy. It is likely that different SCCHN tumors stabilize p62/SQSTM1 and activate Nrf2 through other mechanisms. In fact, recent data demonstrate that some SCCHN harbor activating mutations in Nrf2 [15] suggesting that the p62/SQSTM1-Nrf2 axis could be a prime target for therapy.

Macro-autophagy is known to play a critical role in tissue homeostasis and it has been suggested that tumor cells rely heavily on macro-autophagy for metabolites, energy, and to remove the increased levels of protein aggregates and dysfunctional organelles that accumulate more rapidly in tumor cells [43], [44]. One key avenue of future investigation will therefore be to understand how tumor cells compensate for defects in macro-autophagy in order to degrade cytoplasmic aggregates and organelles. Although macro-autophagy has been well described [44], [45], other forms of autophagy are also known to play a role in cellular housekeeping. Recently, chaperone mediated autophagy (CMA) was shown to be activated in lung and melanoma cell lines and to promote tumor cell growth [46]. Microautophagy involves direct engulfment of cytoplasmic cargo and plays an important role in maintaining nuclear and organelle function. However, whether microautophagy can compensate for deficient macro-autophagy and its broader role in cancer has not been examined. Nonetheless, if cancer cells that are macro-autophagy deficient are more dependent on other forms of autophagy, such as CMA or microautophagy for survival, [47], then these compensating pathways may represent novel therapeutic targets in cancer.

If macro-autophagy does emerge as a meaningful readout of therapeutic efficacy in cancers as our work would suggest, predictive biomarkers of autophagy efficiency will be essential. Here we show that expression of specific autophagy related proteins are associated with sensitivity to PI3K pathway inhibitors. However, measuring autophagy competence or autophagic flux *in situ* in tumors is more complex. Like any dynamic process, levels of autophagy will be subject to change in response to various temporal, environmental, and physiological stresses. Finding the optimal set of biomarkers has proven challenging. While electron microscopy has been considered the gold standard for evaluating autophagy *in situ*, its application to the clinical situation is logistically difficult [48]. Notwithstanding, the data here suggest that p62/SQSTM1, LC3-II, ATG7, and potentially others are candidate pharmacodynamic markers for drugs, including PI3K pathway inhibitors that exert their effects through modulating autophagy.

While autophagy was once considered a form of programmed cell death, it is now recognized that this process functions as an important survival mechanism during cellular stress. The finding that autophagy can be defective in cancer cells and a compromised ability to undergo autophagy is mechanistically related to therapeutic resistance must now be integrated into our understanding of this process in malignant disease and tested in clinical trials. These results, of course, open new avenues of exploration and exploitation of specific phenotypes in the development of novel therapies.

## Supporting Information

File S1 Figure S1. A) MK-2206 and SAR245408 inhibit AKT and S6 phosphorylation. B) MK-2206 and SAR245408 efficiently induce G1-arrest in SCC61 cells but not in SCC35 cells. Figure S2. Effect of MK-2206 and SAR245408 on AKT phosphorylation and cyclin D1 protein in SCCHN cell lines. Figure S3. Alterations of *ATG7* gene in the TCGA Squamous Cell Carcinoma of Head and Neck (SCCHN) database and overall survival. Table S1. IC_50_ of MK-2206 and SAR245408 in SCCHN cell lines. Table S2. Apoptotic assay after 48 hours of treatment with MK02206 or SAR245408. The values are expressed as percentage of total cell number counted. Table S3. Intrinsic autophagy competence determined by LC3-II to LC3-I ratio and IC_50_ of respective agents in SCCHN cell lines. Table S4. Relationship between p62/SQSTM1 protein expression and IC_50_ in SCCHN cell lines. Pearson rank correlation was used to calculate correlation. Table S5. Relationship between p62/SQSTM1 protein expression and IC_50_ in breast cancer cell lines. Pearson rank correlation was used to calculate correlation. Table S6. Primer sequences for *ATG7* MSP analysis.
(DOC)

## References

[1] Fresno Vara JA, Casado E, de Castro J, Cejas P, Belda-Iniesta C, et al (2004) PI3K/Akt signalling pathway and cancer. Cancer Treat Rev 30: 193–204. 15023437 10.1016/j.ctrv.2003.07.007

[2] Janku F, Tsimberidou AM, Garrido-Laguna I, Wang X, Luthra R, et al (2011) PIK3CA mutations in patients with advanced cancers treated with PI3K/AKT/mTOR axis inhibitors. Mol Cancer Ther 10: 558–565. 21216929 10.1158/1535-7163.MCT-10-0994 PMC3072168

[3] Cohen Y, Goldenberg-Cohen N, Shalmon B, Shani T, Oren S, et al (2011) Mutational analysis of PTEN/PIK3CA/AKT pathway in oral squamous cell carcinoma. Oral Oncol 47: 946–950. 21824802 10.1016/j.oraloncology.2011.07.013

[4] Liu P, Cheng H, Roberts TM, Zhao JJ (2009) Targeting the phosphoinositide 3-kinase pathway in cancer. Nat Rev Drug Discov 8: 627–644. 19644473 10.1038/nrd2926 PMC3142564

[5] Degtyarev M, De Maziere A, Orr C, Lin J, Lee BB, et al (2008) Akt inhibition promotes autophagy and sensitizes PTEN-null tumors to lysosomotropic agents. J Cell Biol 183: 101–116. 18838554 10.1083/jcb.200801099 PMC2557046

[6] Janku F, McConkey DJ, Hong DS, Kurzrock R (2011) Autophagy as a target for anticancer therapy. Nature reviews Clinical oncology 8. 10.1038/nrclinonc.2011.71 21587219

[7] Ghadimi MP, Lopez G, Torres KE, Belousov R, Young ED, et al (2012) Targeting the PI3K/mTOR axis, alone and in combination with autophagy blockade, for the treatment of malignant peripheral nerve sheath tumors. Mol Cancer Ther 11: 1758–1769. 22848094 10.1158/1535-7163.MCT-12-0015 PMC3416967

[8] Hunter KD, Parkinson EK, Harrison PR (2005) Profiling early head and neck cancer. Nat Rev Cancer 5: 127–135. 15685196 10.1038/nrc1549

[9] Saman D (2012) A review of the epidemiology of oral and pharyngeal carcinoma: update. Head & Neck Oncology 4: 1. 22244087 10.1186/1758-3284-4-1 PMC3292826

[10] Simard EP, Ward EM, Siegel R, Jemal A (2012) Cancers with increasing incidence trends in the United States: 1999 through 2008. CA: A Cancer Journal for Clinicians 62: 118–128.22281605

[11] Lui VW, Hedberg ML, Li H, Vangara BS, Pendleton K, et al (2013) Frequent Mutation of the PI3K Pathway in Head and Neck Cancer Defines Predictive Biomarkers. Cancer Discov 3: 761–769. 23619167 10.1158/2159-8290.CD-13-0103 PMC3710532

[12] Agrawal N, Frederick MJ, Pickering CR, Bettegowda C, Chang K, et al (2011) Exome sequencing of head and neck squamous cell carcinoma reveals inactivating mutations in NOTCH1. Science 333: 1154–1157. 21798897 10.1126/science.1206923 PMC3162986

[13] Stransky N, Egloff AM, Tward AD, Kostic AD, Cibulskis K, et al (2011) The mutational landscape of head and neck squamous cell carcinoma. Science 333: 1157–1160. 21798893 10.1126/science.1208130 PMC3415217

[14] Keck Michaela K, Zuo Zhixiang, Khattri Arun, Brown Christopher D, Stricker Thomas, et al (2013) Genomic profiling of kinase genes in head and neck squamous cell carcinomas to identify potentially targetable genetic aberrations in FGFR1/2, DDR2, EPHA2, and PIK3CA. J Clin Oncol 31.

[15] Hayes JRGaAKE-N David N (2013) The Cancer Genome Atlas: Integrated analysis of genome alterations in squamous cell carcinoma of the head and neck. J Clin Oncol 31.

[16] Shanware NP, Bray K, Abraham RT (2013) The PI3K, metabolic, and autophagy networks: interactive partners in cellular health and disease. Annu Rev Pharmacol Toxicol 53: 89–106. 23294306 10.1146/annurev-pharmtox-010611-134717

[17] Klionsky DJ (2007) Autophagy: from phenomenology to molecular understanding in less than a decade. Nature reviews Molecular cell biology 8: 931–937. 17712358 10.1038/nrm2245

[18] Parzych KR, Klionsky DJ (2013) An Overview of Autophagy: Morphology, Mechanism, and Regulation. Antioxid Redox Signal 10.1089/ars.2013.5371 PMC3894687 23725295

[19] Sridhar S, Botbol Y, Macian F, Cuervo AM (2011) Autophagy and Disease: always two sides to a problem. The Journal of pathology 255–273. 10.1002/path.3025 PMC3996449 21990109

[20] Rubinsztein DC, Codogno P, Levine B (2012) Autophagy modulation as a potential therapeutic target for diverse diseases. Nat Rev Drug Discov 11: 709–730. 22935804 10.1038/nrd3802 PMC3518431

[21] Cowan JM, Beckett MA, Ahmed-Swan S, Weichselbaum RR (1992) Cytogenetic evidence of the multistep origin of head and neck squamous cell carcinomas. J Natl Cancer Inst 84: 793–797. 1573667 10.1093/jnci/84.10.793

[22] Easty DM, Easty GC, Carter RL, Monaghan P, Butler LJ (1981) Ten human carcinoma cell lines derived from squamous carcinomas of the head and neck. Br J Cancer 43: 772–785. 7195729 10.1038/bjc.1981.115 PMC2010719

[23] Kuo W-L, Liu J, Mauceri H, Vokes EE, Weichselbaum R, et al (2010) Efficacy of the multi-kinase inhibitor enzastaurin is dependent on cellular signaling context. Molecular cancer therapeutics 9: 2814–2824. 20876745 10.1158/1535-7163.MCT-10-0352 PMC2953602

[24] Lee IH, Cao L, Mostoslavsky R, Lombard DB, Liu J, et al (2008) A role for the NAD-dependent deacetylase Sirt1 in the regulation of autophagy. Proc Natl Acad Sci U S A 105: 3374–3379. 18296641 10.1073/pnas.0712145105 PMC2265142

[25] Young NR, Liu J, Pierce C, Wei TF, Grushko T, et al (2013) Molecular phenotype predicts sensitivity of squamous cell carcinoma of the head and neck to epidermal growth factor receptor inhibition. Mol Oncol 7: 359–368. 23200321 10.1016/j.molonc.2012.11.001 PMC3661759

[26] Hennessy BT, Smith DL, Ram PT, Lu Y, Mills GB (2005) Exploiting the PI3K/AKT pathway for cancer drug discovery. Nat Rev Drug Discov 4: 988–1004. 16341064 10.1038/nrd1902

[27] Sangai T, Akcakanat A, Chen H, Tarco E, Wu Y, et al (2012) Biomarkers of Response to Akt Inhibitor MK-2206 in Breast Cancer. Clin Cancer Res 18: 5816–5828. 22932669 10.1158/1078-0432.CCR-12-1141 PMC3772348

[28] Lee HS, Daniels BH, Salas E, Bollen AW, Debnath J, et al (2012) Clinical utility of LC3 and p62 immunohistochemistry in diagnosis of drug-induced autophagic vacuolar myopathies: a case-control study. PLoS One 7: e36221. 22558391 10.1371/journal.pone.0036221 PMC3338695

[29] Puissant A, Fenouille N, Auberger P (2012) When autophagy meets cancer through p62/SQSTM1. Am J Cancer Res 2: 397–413. 22860231 PMC3410580

[30] Jaakkola PM, Pursiheimo JP (2009) p62 degradation by autophagy: another way for cancer cells to survive under hypoxia. Autophagy 5: 410–412. 19197142 10.4161/auto.5.3.7823

[31] Ravikumar B, Sarkar S, Davies JE, Futter M, Garcia-arencibia M, et al (2010) Regulation of Mammalian Autophagy in Physiology and Pathophysiology. Physiological Reviews 1383–1435. 20959619 10.1152/physrev.00030.2009

[32] Klionsky DJ, Abdalla FC, Abeliovich H, Abraham RT, Acevedo-Arozena A, et al (2012) Guidelines for the use and interpretation of assays for monitoring autophagy. Autophagy 8: 445–544. 22966490 10.4161/auto.19496 PMC3404883

[33] Bjørkøy G, Lamark T, Brech A, Outzen H, Perander M, et al (2005) p62/SQSTM1 forms protein aggregates degraded by autophagy and has a protective effect on huntingtin-induced cell death. The Journal of cell biology 171: 603–614. 16286508 10.1083/jcb.200507002 PMC2171557

[34] Fujita K, Maeda D, Xiao Q, Srinivasula SM (2011) Nrf2-mediated induction of p62 controls Toll-like receptor-4-driven aggresome-like induced structure formation and autophagic degradation. Proc Natl Acad Sci U S A 108: 1427–1432. 21220332 10.1073/pnas.1014156108 PMC3029726

[35] Kwon J, Han E, Bui CB, Shin W, Lee J, et al (2012) Assurance of mitochondrial integrity and mammalian longevity by the p62-Keap1-Nrf2-Nqo1 cascade. EMBO Rep 13: 150–156. 22222206 10.1038/embor.2011.246 PMC3271336

[36] Lau A, Wang XJ, Zhao F, Villeneuve NF, Wu T, et al (2010) A noncanonical mechanism of Nrf2 activation by autophagy deficiency: direct interaction between Keap1 and p62. Mol Cell Biol 30: 3275–3285. 20421418 10.1128/MCB.00248-10 PMC2897585

[37] Pankiv S, Lamark T, Bruun JA, Øvervatn A, Bjørkøy G, et al (2010) Nucleocytoplasmic shuttling of p62/SQSTM1 and its role in recruitment of nuclear polyubiquitinated proteins to promyelocytic leukemia bodies. J Biol Chem 285: 5941–5953. 20018885 10.1074/jbc.M109.039925 PMC2820819

[38] Cerami E, Gao J, Dogrusoz U, Gross BE, Sumer SO, et al (2012) The cBio cancer genomics portal: an open platform for exploring multidimensional cancer genomics data. Cancer Discov 2: 401–404. 22588877 10.1158/2159-8290.CD-12-0095 PMC3956037

[39] Degenhardt K, Mathew R, Beaudoin B, Bray K, Anderson D, et al (2006) Autophagy promotes tumor cell survival and restricts necrosis, inflammation, and tumorigenesis. Cancer Cell 10: 51–64. 16843265 10.1016/j.ccr.2006.06.001 PMC2857533

[40] Takamura A, Komatsu M, Hara T, Sakamoto A, Kishi C, et al (2011) Autophagy-deficient mice develop multiple liver tumors. Genes Dev 25: 795–800. 21498569 10.1101/gad.2016211 PMC3078705

[41] Yu L, Alva A, Su H, Dutt P, Freundt E, et al (2004) Regulation of an ATG7-beclin 1 program of autophagic cell death by caspase-8. Science 304: 1500–1502. 15131264 10.1126/science.1096645

[42] Lee IH, Kawai Y, Fergusson MM, Rovira II, Bishop AJ, et al (2012) Atg7 modulates p53 activity to regulate cell cycle and survival during metabolic stress. Science 336: 225–228. 22499945 10.1126/science.1218395 PMC4721513

[43] Mathew R, Karp CM, Beaudoin B, Vuong N, Chen G, et al (2009) Autophagy suppresses tumorigenesis through elimination of p62. Cell 137: 1062–1075. 19524509 10.1016/j.cell.2009.03.048 PMC2802318

[44] White E (2012) Deconvoluting the context-dependent role for autophagy in cancer. Nat Rev Cancer 12: 401–410. 22534666 10.1038/nrc3262 PMC3664381

[45] Bialik S, Kimchi A (2008) Autophagy and tumor suppression: recent advances in understanding the link between autophagic cell death pathways and tumor development. Adv Exp Med Biol 615: 177–200. 18437896 10.1007/978-1-4020-6554-5_9

[46] Kon M, Kiffin R, Koga H, Chapochnick J, Macian F, et al (2011) Chaperone-Mediated Autophagy Is Required for Tumor Growth. Science translational medicine 3: 109ra117. 10.1126/scitranslmed.3003182 PMC4000261 22089453

[47] Kaushik S, Massey AC, Mizushima N, Cuervo AM (2008) Constitutive Activation of Chaperone-mediated Autophagy in Cells with Impaired Macroautophagy. Molecular Biology of the Cell 19: 2179–2192. 18337468 10.1091/mbc.E07-11-1155 PMC2366850

[48] Ma XH, Piao S, Wang D, McAfee QW, Nathanson KL, et al (2011) Measurements of tumor cell autophagy predict invasiveness, resistance to chemotherapy, and survival in melanoma. Clin Cancer Res 17: 3478–3489. 21325076 10.1158/1078-0432.CCR-10-2372 PMC3096713

