# Supplementary material for: p62/SQSTM1 Accumulation in Squamous Cell Carcinoma of Head and Neck Predicts Sensitivity to Phosphatidylinositol 3-Kinase Pathway Inhibitors
Source: PLoS One. 2014 Mar 5;9(3):e90171. doi: 10.1371/journal.pone.0090171 (PMC3943907; doi:10.1371/journal.pone.0090171)

**Supporting Information**

***Supplementary Tables***

**Table S1:** IC_50_ of MK-2206 and SAR245408 in SCCHN cell lines. SEM = standard error of the mean.

| **Cell Line** | **MK-2206** | | **SAR245408** | |
| --- | --- | --- | --- | --- |
|  | **IC50 (µM)** | **SEM** | **IC50 (µM)** | **SEM** |
| **SCC61** | **3.6** | **0.9** | **8.5** | **0.5** |
| **SCC58** | **4.2** | **0.6** | **3.0** | **0.7** |
| **SCC28** | **4.8** | **0.4** | **6.6** | **0.7** |
| **SCC25** | **5.3** | **0.3** | **5.6** | **0.5** |
| **CAL27** | **6.2** | **1.5** | **10.5** | **1.4** |
| **SCC35** | **6.8** | **1.6** | **13.5** | **1.7** |
| **Detroit 562** | **7.6** | **0.5** | **12.6** | **1.3** |

**Table S2:** Apoptotic assay after 48 hours of treatment with MK02206 or SAR245408. The values are expressed as percentage of total cell number counted.

| **Cell Line** | **Treatment)** | **Viable Cells** | **Early apoptotic** | **Late apoptotic** | **Dead or necrotic** |
| --- | --- | --- | --- | --- | --- |
| **SCC61** | **Control** | **93.45 ± 0.15** | **0.20 ± 0.04** | **0.28 ± 0.03** | **6.05 ± 0.16** |
|  | **MK-2206** | **90.45 ± 0.35** | **0.48 ± 0.12** | **1.13 ± 0.37** | **7.95 ± 0.79** |
|  | **SAR245408** | **89.20 ± 0.90** | **0.46 ± 0.01** | **1.43 ± 0.07** | **8.92 ± 0.89** |
| **SCC35** | **Control** | **93.55 ± 1.35** | **0.67 ± 0.02** | **0.95 ± 0.03** | **4.81 ± 1.31** |
|  | **MK-2206** | **92.70 ± 0.10** | **1.16 ± 0.01** | **2.31 ± 0.24** | **3.83 ± 0.36** |
|  | **SAR245408** | **90.05 ± 0.05** | **1.74 ± 0.03** | **2.08 ± 0.30** | **6.14 ± 0.29** |

**Table S3:** Intrinsic autophagy competence determined by LC3-II to LC3-I ratio and IC_50_ of respective agents in SCCHN cell lines.

| **Cell Line** | **LC-3II/LC3-I**  **(Ratio)** | **MK-2206 (µM)** | **SAR245408 (µM)** |
| --- | --- | --- | --- |
| **SCC58** | **9.0** | **4.2** | **3.0** |
| **SCC61** | **7.1** | **3.6** | **8.5** |
| **CAL27** | **4.2** | **6.2** | **10.5** |
| **SCC25** | **1.9** | **5.3** | **5.6** |
| **SQ20B** | **1.9** | **7.8** | **8.5** |
| **SCC15** | **1.6** | **5.2** | **8.5** |
| **Detroit 562** | **1.2** | **7.6** | **12.6** |
| **SCC35** | **0.0** | **6.9** | **13.5** |

**Table S4:** Relationship between p62/SQSTM1 protein expression and IC_50_ in SCCHN cell lines**.** Pearson rank correlation was used to calculate correlation.

| **Cell Line** | | **P62/SQSTM1 (arbitrary unit)** | **MK-2206 (µM)** | **SAR245408 (µM)** |
| --- | --- | --- | --- | --- |
| **SCC35** | **173** | | **6.88** | **13.69** |
| **CAL27** | **152** | | **6.20** | **11.17** |
| **Detroit 562** | **124** | | **7.61** | **12.87** |
| **SCC28** | **102** | | **4.79** | **6.56** |
| **SCC61** | **101** | | **3.61** | **8.56** |
| **SCC58** | **80** | | **4.20** | **6.03** |
| **SCC25** | **53** | | **5.30** | **5.57** |
|  | | | | |
| **Pearson r** |  | | **0.61** | **0.82** |
| **P (one-tailed)** |  | | **0.07** | **0.01** |
| **P Summary** |  | | **ns** | ***** |

**Table S5:** Relationship between p62/SQSTM1 protein expression and IC_50_ in breast cancer cell lines. Pearson rank correlation was used to calculate correlation.

| **Cell Line** | | **P62/SQSTM1 (arbitrary unit)** | **MK-2206 (µM)** | **SAR245408 (µM)** |
| --- | --- | --- | --- | --- |
| **HCC38** | **138.6** | | **16.3** | **48.1** |
| **HCC1937** | **125.2** | | **9.6** | **85.2** |
| **MDA-MB231** | **33.0** | | **10.3** | **19.8** |
| **T47D** | **22.5** | | **0.3** | **12.3** |
| **MDA-MB468** | **18.4** | | **1.9** | **8.6** |
| **HS578T** | **13.5** | | **13.9** | **22.9** |
| **MCF7** | **7.3** | | **0.6** | **8.4** |
| **SKBR3** | **4.3** | | **0.6** | **7.0** |
|  | | | | |
| **Pearson r** |  | | **0.72** | **0.89** |
| **P (one-tailed)** |  | | **0.022** | **0.001** |
| **P Summary** |  | | ***** | ******* |

**Table S6:** Primer sequences for *ATG7* MSP analysis

**Primer sequence (5’ - 3’)**

**M Forward TGT GTT GCG TTT GAT GTC**

**Reverse CCC GAA AAA AAC GAT AAA AA**

**U Forward TTT TGT GTT GTG TTT GAT GTT**

**Reverse CCC CCA AAA AAA ACA ATA AAA AA**

**W Forward TGT GCT GCG TTT GAT GCC**

**Reverse CCC GGG GGA AGC GAT GGA GA**

***Supplementary Figure Legends and Figures***

**Figure S1 A)** MK-2206 and SAR245408 inhibit AKT and S6 phosphorylation. Cells were treated with enzastaurin (10 µM,), MK-2206 (5 µM), SAR245408 (10 µM), or SAR245409 (10 µM) for 24 hours. The lysates were analyzed by western blotting and probed with the antibodies as indicated. **B)** MK-2206 and SAR245408 efficiently induce G1-arrest in SCC61 cells but not in SCC35 cells. Cells were serum-starved for 24 hours and treated with MK-2206 (5 µM) or SAR245408 (10 µM) for 24 hours. The cells were stained with Propidium Iodide for cell cycle profiling by flow cytometry as described in Methods.

**Figure S2** Effect of MK-2206 and SAR245408 on AKT phosphorylation and cyclin D1 protein in SCCHN cell lines. In-cell western analysis of phospho-AktS473 and cyclin D1 protein expression in SCCHN cells treated with MK2206 (5 µM) and SAR245408 (10 µM) Cells were seeded in 96-wells, serum-starved, and treated with MK2206 and SAR245408 for the times indicated. Cells were fixed in 4% formaldehyde, washed, and incubated with anti-phospho-AKT **A)** and cyclin D1 **B)** antibodies, separately. DNA-staining dye, TO-PRO-3, was used for estimating cell numbers in each well. The Odyssey Infrared Imaging System was used for scanning and quantifying the results. The values of pAKT-S473 and cyclin D1 were normalized to the cell numbers in each well. Each determinant was calculated from triplicate wells.

**Figure S3** Alterations of *ATG7* gene in the TCGA Squamous Cell Carcinoma of Head and Neck (SCCHN) database and overall survival. **A)** The OncoPrint of *ATG7* gene in the SCCHN TCGA database. Individual samples are represented as columns and alterations of homozygous deletion (blue), ATG7 mRNA down-regulation (green), and up-regulation (orange) are indicated. **B)** Overall survival analysis. Cases with one or two *ATG7* alterations have worse overall survival than cases without an *ATG7* gene alteration. The data were obtained and visualized by cBioPortal (<http://www.cbioportal.org/public-portal/>) using the TCGA Head and Neck Squamous Cell Carcinoma data set.

**Figure S4** Ectopic expression of ATG7 to rescue autophagy in SCC35 cells. Western blots show **A)** the expression of mycAtg7 in SCC35 cells and endogenous p62/SQSTM1 by sequential probing of the membrane with Atg7 antibody and p62/SQSTM1 antibody. Lighter exposure of the p62/SQSTM1 bands are shown in **B)** The same membrane was then probed with Atg5 antibody to detect Atg5/Atg12 conjugates and free form of Atg5 in **C)** Ectopic expression of Atg7 in SCC35 did not change LC3 conversion or drug sensitivity to MK-2206. **D)** Detection of LC3-I by western blotting in the parental and myc-Atg7 expressing SCC35 cells. **E)** Dose responsive curves of SCC35 and SCC35-myc-Atg7 cell lines to MK-2206.

**Figure S1**

**
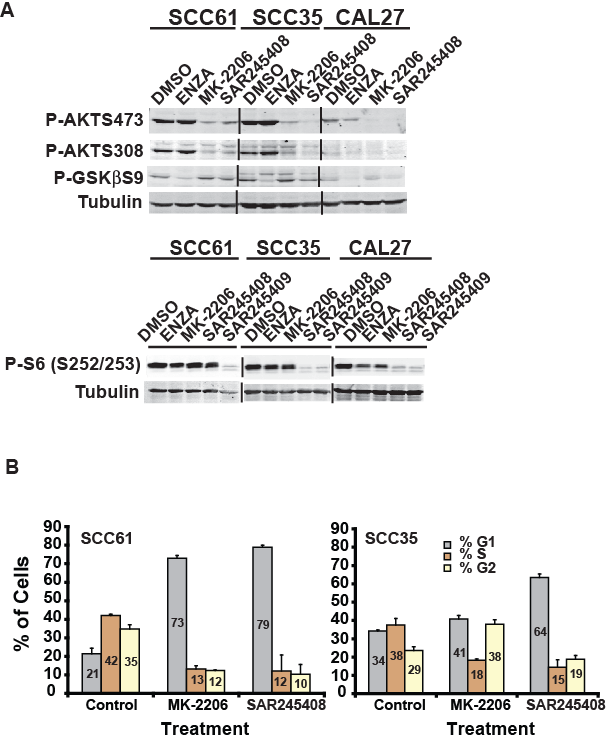
**


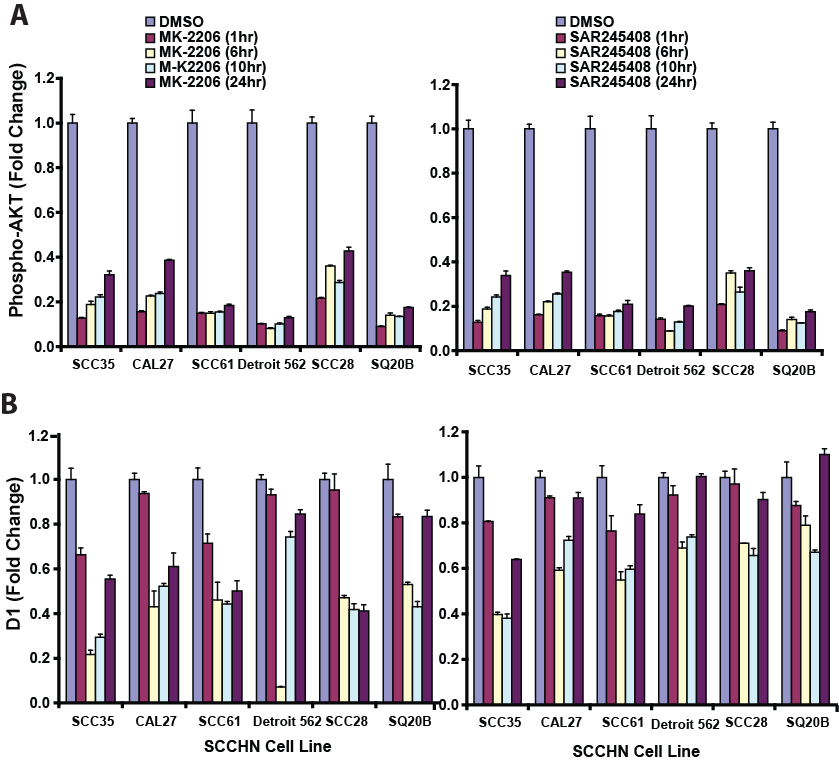
**Figure S2**

**Figure S3**

**
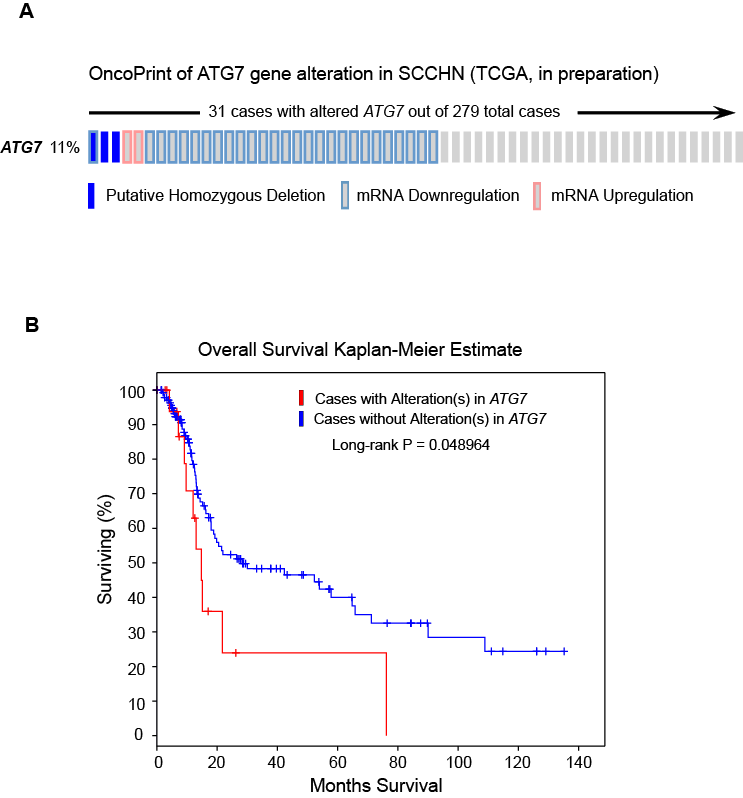
**

**Figure S4**


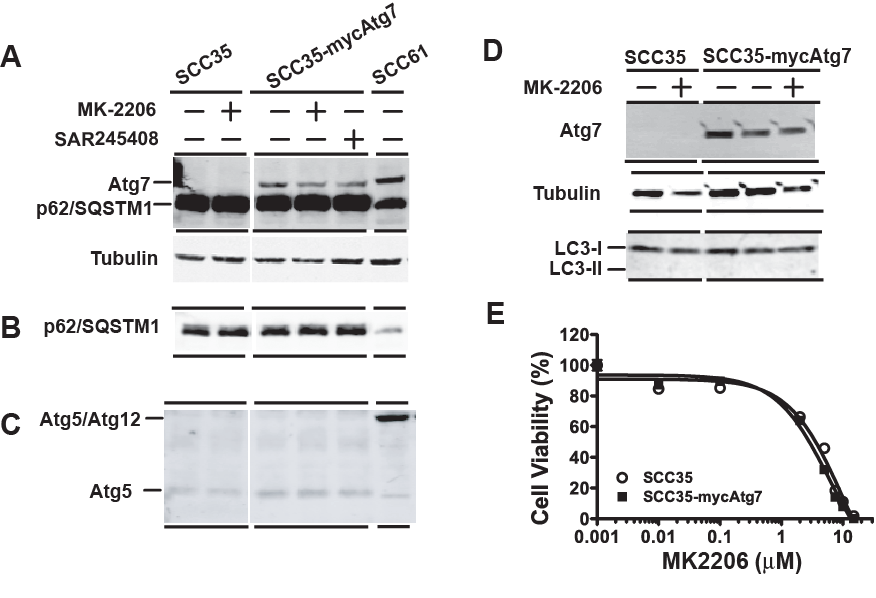

Supplement: File S1 — Figure S1. A) MK-2206 and SAR245408 inhibit AKT and S6 phosphorylation. B) MK-2206 and SAR245408 efficiently induce G1-arrest in SCC61 cells but not in SCC35 cells. Figure S2. Effect of MK-2206 and SAR245408 on AKT phosphorylation and cyclin D1 protein in SCCHN cell lines. Figure S3. Alterations of ATG7 gene in the TCGA Squamous Cell Carcinoma of Head and Neck (SCCHN) database and overall survival. Table S1. IC50 of MK-2206 and SAR245408 in SCCHN cell lines. Table S2. Apoptotic assay after 48 hours of treatment with MK02206 or SAR245408. The values are expressed as percentage of total cell number counted. Table S3. Intrinsic autophagy competence determined by LC3-II to LC3-I ratio and IC50 of respective agents in SCCHN cell lines. Table S4. Relationship between p62/SQSTM1 protein expression and IC50 in SCCHN cell lines. Pearson rank correlation was used to calculate correlation. Table S5. Relationship between p62/SQSTM1 protein expression and IC50 in breast cancer cell lines. Pearson rank correlation was used to calculate correlation. Table S6. Primer sequences for ATG7 MSP analysis. (DOC) [file pone.0090171.s001.doc]
